# An innovative fixation technique by osteosuture in a young athletic female patient with coracoid process fracture: a case report

**DOI:** 10.11604/pamj.2021.40.218.30731

**Published:** 2021-12-11

**Authors:** Juliette Fradet, Alexandre Losson, Christopher Koneazny, Tanguy Vendeuvre

**Affiliations:** 1 Department of Orthopaedic Surgery, Orthopaedic Unit, University Hospital Poitiers, Paris, France

**Keywords:** Fracture avulsion, shoulder injuries, fracture fixation, coracoclavicular joint, case report

## Abstract

Coracoid process fractures are uncommon lesions but are frequently associated with an acromioclavicular dislocation. The aim of this article is to report our experience of an innovative fixation technique by osteosuture in a young athletic female patient presenting a displaced fracture of the coracoid process on the insertion footprint of coracoclavicular ligaments, with no breach of continuity of the lower coracoid cortex. She also had a roockwood type 3 acromioclavicular dislocation. After a deltopectoral approach, the fracture was reduced to the anatomical position and stabilized by a first lacing, using a non-absorbable large caliber thread passing under the mid part of the coracoid process between the 2 coracoclavicular ligaments. A second lacing passing under the coracoid process and through the trapezoid ligament, and a third one through the conoïd ligament. At 6 months, the fracture was consolidated and the constant score was 100/100. This innovative fixation provides good clinical and radiological results in the short and medium term.

## Introduction

Fractures of the coracoid process are uncommon lesions that may occur either isolated or associated with other shoulder injuries. The association with an acromioclavicular dislocation (ACD) is the most frequently seen [1]. The diagnosis is difficult, often overlooked on standard X-rays and requires a morphological assessment by computed tomography. Without treatment, a coracoid process (CP) fracture will lead to chronic pain and functional impairment. The most common surgical management is an osteosynthesis by screw fixation. It enables to get a good bone consolidation and the patient´s satisfaction. The main aim of this case report is to share our experience of a fixation technique of osteosuture by lacing of a coracoid process avulsion fracture of the coracoclavicular ligaments insertion, associated with an acromioclavicular dislocation.

## Patient and observation

The patient is a 23-year-old woman with acute pain and functional impairment of the left shoulder due to a fall from a horse. The patient is a right handed, sporty nursing student, with no medical history relating to her left shoulder.

**Clinical findings:** the clinical examination found a “piano key-shaped” deformity with vertical instability of the acromioclavicular joint, without anteroposterior instability. No clinical signs of brachial plexus or vascular injury were found.

**Figure 1:**
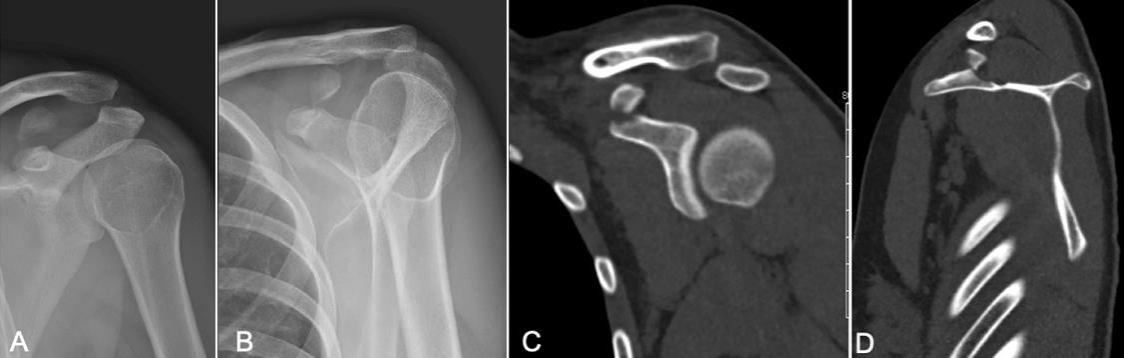
preoperative imaging, A) AP X-rays; B) lateral X-rays; C) CT-scan coronal view; D) and sagittal view

**Diagnosis assessment:** the initial morphological assessment found a type 3 ACD and a displaced fracture of the CP at the coracoclavicular ligaments insertion with no breach to the lower coracoid cortex (Figure 1). This type of fracture is not precisely described in the commonly used classifications.

**Figure 2:**
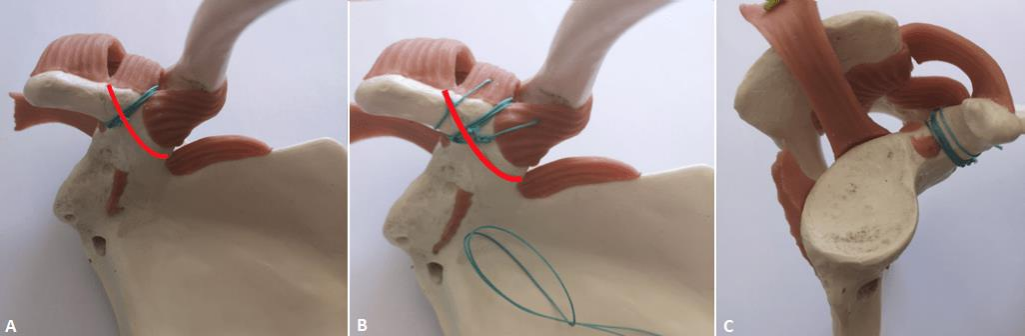
prototype representation of the fracture line (in red) and of the 3 lacings performed, A) AP view of the first lacing; B) AP view with representation of a “gull winged” node; C) and lateral view

**Therapeutic intervention:** a classic osteosynthesis by screw fixation would have been difficult given the orientation of the fracture line. Twenty four (24) hours after her trauma, the patient was operated under general anesthesia, in a beach chair position. The first surgical step was the reduction of the ACD by performing an open acromioclavicular arthrorisis with 2 K-wires, associated with a suture of the acromioclavicular ligament. The second step was an osteosuture of the fracture using a deltopectoral approach. The coracoclavicular ligaments were intact. The fracture was reduced to the anatomical position and stabilized by a first lacing with a Mersuture* 3 surgical thread, passing under the mid part of the CP between the 2 coracoclavicular ligaments. A second lacing passing under the CP and through the trapezoid ligament, and a third lacing through the conoid ligament. An intraoperative testing confirmed the osteosuture´s stability. We represented on a prototype the fracture as it was observed during the surgery, and the three lacings with one Mersuture* 3 thread on the same prototype. The first “gull-winged” lacing between the 2 coracoclavicular ligaments is perpendicular to the fracture line, and the 2 other lacings are trans ligamentous (Figure 2).

**Figure 3:**
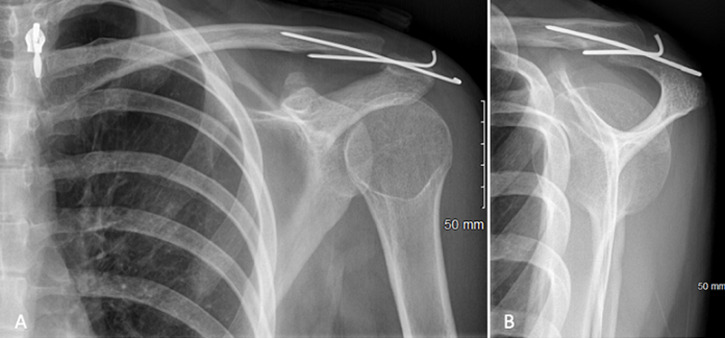
A) post-operative X-rays on day 1, AP; B) and lateral view

**Figure 4:**
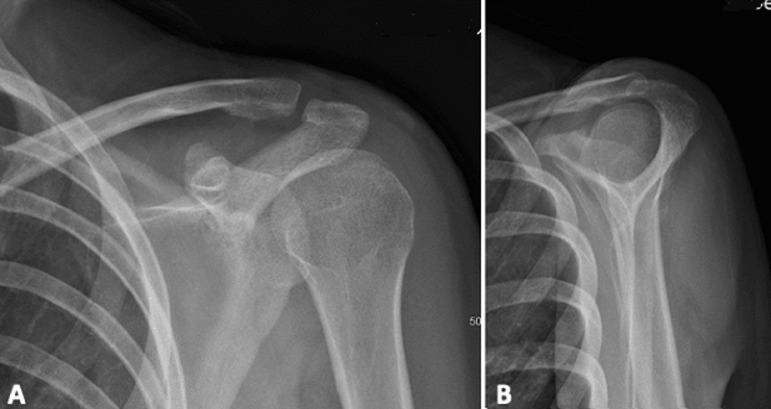
A) post-operative X-rays on day 45, AP; B) and lateral view

**Figure 5:**
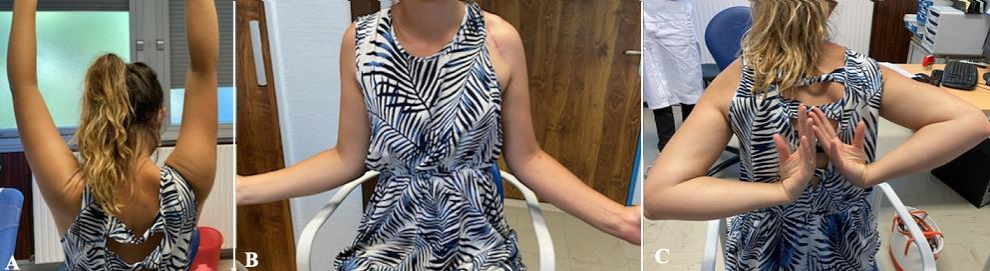
bilateral active mobility 3 months postoperatively, A) flexion; B) external rotation; C) internal rotation

**Figure 6:**
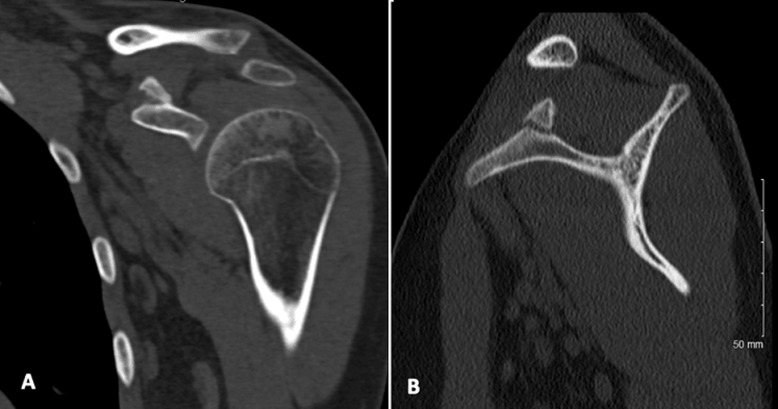
CT-scan views 3 months post operatively, A) coronal view; B) sagittal view

**Figure 7:**
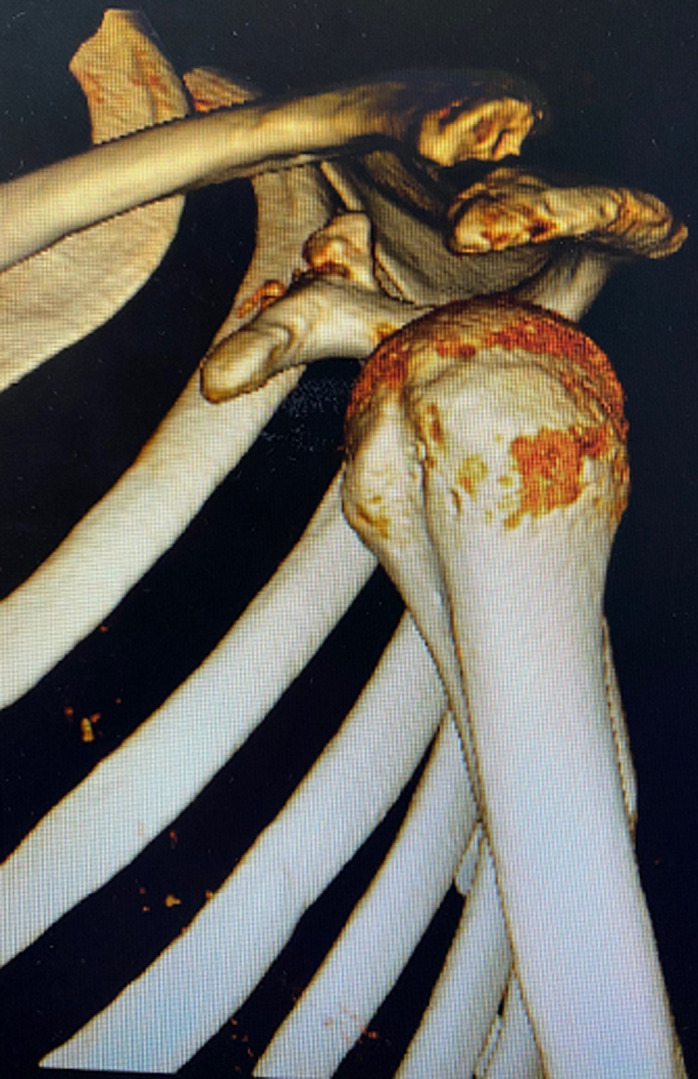
three-dimensional reconstruction showing the reduction and the callus of the coracoid process at 6 months

**Follow-up and outcomes:** postoperatively the patient was immobilized in a shoulder brace for 6 weeks, passive range of motion was authorized immediately after surgery and X-rays control (Figure 3). Acromioclavicular arthrorisis pins were removed at 6 weeks without any complications (Figure 4). The clinical examination (Figure 5) at 3 months found a constant score of 92/100 (active mobility 40/40, pain 15/15, daily activities 12/20, strength 25/25). The imaging checkup at 3 months (X-rays and computerized tomography) showed a partially consolidated fracture with a satisfactory reduction associated with a bone callus allowing the resumption of sports activities (Figure 6). At 6 months, bone consolidation was fully attained (Figure 7), and the patient had completely gone back to her previous professional and sport activities (constant score of 100/100).

**Patient perspective:** during the time she was hospitalized and after the treatment, the patient was delighted with the care she received and was optimistic about the outcome of her condition.

**Informed consent:** the patient was informed of the case report, why her case was unique, and the authors' interest in publishing her case.

**Patient consent:** she has given her consent for her images and other clinical informations to be reported in the journal. The patient understands that her name and initials will not be published.

## Discussion

Patient management is different between conservative and surgical treatment, and there are no clear recommendations regarding these injuries. The majority of isolated coracoid fractures can be treated conservatively when there is no or little displacement [2,3]. Surgery is recommended when there is a fracture displacement greater than 1cm, or an associated fracture of the scapula, or associated lesions of the shoulder girdle [4,5] and in patients who are at increased risk from non-union. Robinson [6] described an association between coracoid fracture nonunion and patients with seizure disorders in five patients; all five patient was operated. In our case, the patient had a lesion of the shoulder girdle associated with a fracture of the CP. The surgical indication was therefore legitimate. Moreover, patients who are involved in competitive athletics or heavy manual labor are at increased risk for non-union when the management is non-operative [2]. Our patient used to ride horses at a high level.

There are multiple approaches to open reduction and internal fixation (ORIF) of the coracoid process that have been described in the literature, including interfragmentary screw fixation for large fragments [7-9] and sutures for small fragments [10]. The interest of our case is the original fixation technique, non-previously described in the literature, by an osteosuture of the CP fracture enabling its reduction and stabilization. In 2017, Kennedy *et al*. described a new surgical technique using an anchor instead of a screw fixation via a deltopectoral approach [11]. The results of their technique at 3 months was satisfactory, with bone consolidation. Yet, the clinical results were not precisely described. In 2020, Passaplan *et al*. describe an arthroscopic screw fixation technique [12]. The procedure is technically demanding but, it permits a very good visualization of the fracture for reduction and fixation, whereas visualization of the coracoid process remains difficult even in open procedures. The arthroscopic approach is an effective and minimally invasive technique, allowing for rapid rehabilitation while avoiding the deep dissection of the deltopectoral interval and extensive soft-tissue dissection necessary in open procedures to expose the coracoid process.

We couldn't use these techniques because it was impossible to be perpendicular to the fracture site in an open approach; the synthesis would therefore not have been satisfactory regardless of using an anchor or a screw. In our case, the constant score was excellent and went from 92/100 at 3 months (related to the work stoppage and no sport) to 100/100 at 6 months, with very good functional results. The bone was incompletely consolidated at 3 months, but a bone callus was present, and the fracture was still reduced without secondary displacement. Some authors found a constant score between 92 and 95/100 after screw fixation, but only after a 24 to 36 month follow-up [13,14]. We chose to surgically treat the ACD. Although some authors have observed an automatic reduction of the ACD once the CP fracture had been reduced and stabilized [13,15] (involving intact coracoclavicular ligaments), we preferred to protect the osteosuture of the coracoid process. Furthermore, we used K-wires because they bring fewer complications than a plate which will cause discomfort and limit rehabilitation [16,17].

## Conclusion

The technique described is based on the use of lacing instead of screw fixation to reduce a displaced coracoid fracture. It provides good clinical and radiological results in the short and medium term. A larger cohort is needed for the evaluation and validation of this technique.
